# A comparison of Ki-67 counting methods in luminal Breast Cancer: The Average Method vs. the Hot Spot Method

**DOI:** 10.1371/journal.pone.0172031

**Published:** 2017-02-10

**Authors:** Min Hye Jang, Hyun Jung Kim, Yul Ri Chung, Yangkyu Lee, So Yeon Park

**Affiliations:** 1 Department of Pathology, Seoul National University Bundang Hospital, Seongnam, Republic of Korea; 2 Department of Pathology, Yeungnam University Medical Center, Daegu, Republic of Korea; 3 Department of Pathology, Seoul National University College of Medicine, Seoul, Republic of Korea; University of North Carolina at Chapel Hill School of Medicine, UNITED STATES

## Abstract

In spite of the usefulness of the Ki-67 labeling index (LI) as a prognostic and predictive marker in breast cancer, its clinical application remains limited due to variability in its measurement and the absence of a standard method of interpretation. This study was designed to compare the two methods of assessing Ki-67 LI: the average method vs. the hot spot method and thus to determine which method is more appropriate in predicting prognosis of luminal/HER2-negative breast cancers. Ki-67 LIs were calculated by direct counting of three representative areas of 493 luminal/HER2-negative breast cancers using the two methods. We calculated the differences in the Ki-67 LIs (ΔKi-67) between the two methods and the ratio of the Ki-67 LIs (H/A ratio) of the two methods. In addition, we compared the performance of the Ki-67 LIs obtained by the two methods as prognostic markers. ΔKi-67 ranged from 0.01% to 33.3% and the H/A ratio ranged from 1.0 to 2.6. Based on the receiver operating characteristic curve method, the predictive powers of the KI-67 LI measured by the two methods were similar (Area under curve: hot spot method, 0.711; average method, 0.700). In multivariate analysis, high Ki-67 LI based on either method was an independent poor prognostic factor, along with high T stage and node metastasis. However, in repeated counts, the hot spot method did not consistently classify tumors into high vs. low Ki-67 LI groups. In conclusion, both the average and hot spot method of evaluating Ki-67 LI have good predictive performances for tumor recurrence in luminal/HER2-negative breast cancers. However, we recommend using the average method for the present because of its greater reproducibility.

## Introduction

Ki-67 is a well-known nuclear proliferation marker. It is a nuclear non-histone protein that is expressed in all phases of the cell cycle except G0, and can be easily detected by immunohistochemistry [1]. Since proliferation is a hallmark of malignant neoplasms, many researchers have investigated its clinical utility as a prognostic and/or predictive biomarker in breast cancer. The prognostic role of Ki-67 labeling index (LI) has been extensively studied especially in early breast cancers, and its usefulness has been proven [2].

However, the use of Ki-67 LI in everyday practice is not easy. First, although Ki-67 LI is a useful biomarker for differentiating the luminal B subtype from the luminal A subtype of breast cancer, there is no established cutoff point. The 2011 St. Gallen consensus advocated a cutoff value of 14% based on the study by Cheang et al. [3, 4]. However, they changed this recommendation at the 2013 consensus: while most of the panel suggested a threshold of 20% or more for high Ki-67 status, others were concerned about the large inter-laboratory variation in Ki-67 measurements, and recommended that each laboratory set its cutoff value independently [5]. Finally, the 2015 consensus suggested that Ki-67 score should be interpreted in the light of local laboratory values [6]. It is likely that the variation in Ki-67 cutoff values depends on the use of different clinical end points, type of treatment, distribution of cases and analytic methodology [7]. Second, the method of interpretation remains an issue. There is no standardized method of Ki-67 assessment: although the International Ki67 Breast Cancer working group issued assessment guidelines, these guidelines have several limitations and have not been widely accepted [8]. And many studies have demonstrated significant inter-observer and intra-observer variability in the interpretation of Ki-67 LIs [9–13].

Hot spots, which refer to areas with a much higher Ki-67 LI than the surrounding areas, can lead to such intra- and inter-observer variability. The approach to scoring hot spots varies across studies; some investigators have focused specifically on the analysis of hot spots, others have included hot spots in the general assessment of Ki-67 LIs across sections, and yet others recommend avoiding them altogether [8]. The international Ki-67 working group recommended assessing complete sections and recording the average score including hot spots (if present) [8]. However, they did not provide any substantial evidence for this recommendation.

In the current study we compared the two methods of assessing Ki-67 LIs: the hot spot method, scoring Ki-67 LIs in a single hot spot only vs. the average method, scoring Ki-67 LIs in multiple areas including hot spot, to determine which method is more appropriate for assessing the prognoses of luminal/HER2-negative breast cancers.

## Materials and methods

### Ethics statement

This study was approved by the institutional review board of Seoul National University Bundang Hospital (protocol # B-1601/332-304). The requirements for informed consent from participants were waived by the institutional review board as all the specimen were previously collected for pathologic examination after surgery and all the data were analyzed anonymously.

### Case selection

Primary luminal/HER2-negative invasive breast cancers were selected from the pathology archives of Seoul National University Bundang Hospital from June 2003 to December 2009. Consecutive surgical resection specimens were obtained and fixed in 10%-formalin and paraffin embedded. Hormone receptor and HER2 status were evaluated by immunohistochemistry (IHC) in whole sections at the time of diagnosis. Following the 2007 ASCO/CAP guidelines [14], HER2 expression was scored, and breast cancers with 2+ HER2 scores on IHC were re-evaluated by fluorescence in situ hybridization. For estrogen receptor (ER) status, breast cancers with ≥1% positive cells on IHC were considered ER positive following the 2010 ASCO/CAP guidelines [15]. However, some studies have shown that low ER-positive breast cancers, with 1%–10% ER expression, have survival that is intermediate between the historical ER-positive (≥10%) and ER-negative (<1%) groups and are of a different molecular subtype [16, 17]. Therefore, to avoid a bias from low ER positivity, we only included breast cancers with ≥10% ER positivity in the study population. ER and progesterone receptor (PR) expression was scored in 10% increments. Cases that had received neoadjuvant chemotherapy and those with distant metastases at the time of diagnosis were excluded. Finally 493 luminal/HER-2 negative breast cancers were included.

### Immunohistochemical staining of Ki-67

Immunohistochemical staining of Ki-67 was performed with the MIB-1 clone (1:500; DAKO, Carpinteria, CA) on the most representative section of each case at the time of diagnosis or during the study. Briefly, four ųm-thick tissue sections were cut, dried, deparaffinized, and rehydrated following standard procedures. All the sections were subjected to heat-induced antigen retrieval. Immunohistochemical staining was carried out in a BenchMark XT autostainer (Ventana Medical Systems, Tucson, AZ) using an i-View or UltraView detection kit.

To test whether the Ki-67 staining had faded due to prolonged storage, we compared newly stained and old slides from the same case. Although staining intensity had decreased slightly, the counting of positive tumor cells was not affected.

### Interpretation of the Ki-67 LI

**Fig 1:**
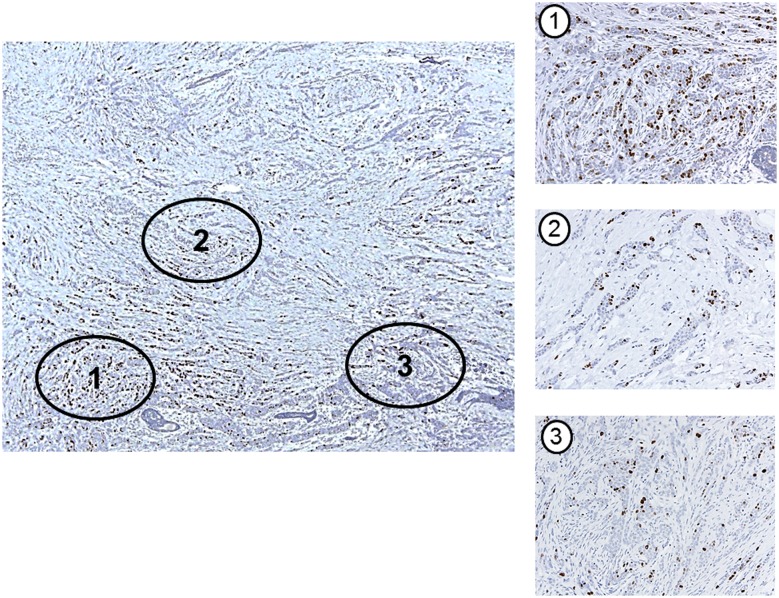
An example of counting area selection in a tumor with heterogeneous Ki-67 expression. Three representative areas have been selected including a hot spot (area 1). (Original magnification: x 40 (large photo); x 200 (three small photos)).

**Fig 2:**
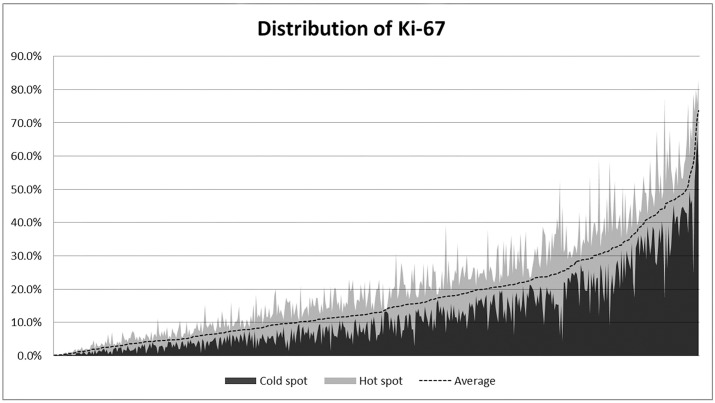
The distribution of Ki-67 labeling indices in 493 luminal breast cancers. The X-axis represents the individual cases, arranged in ascending order based on their Ki-67 labeling index (LI) measured by the average method. The Y-axis represents the individual Ki-67 LIs measured by the hot spot method (the edge of the light gray area) and the average method (dotted line), and the values measured at the “cold spot”, the area with the lowest Ki-67 LI (the edge of the dark gray area). The extent of the light gray colored area represents the difference between the Ki-67 LI measured at the hot spot and the cold spot (Δ Ki-67).

To restrict the area for counting, two pathologists (MHJ and YRC) selected three representative regions from all the sections of each case. Then microphotographs were taken at the same magnification (200x). Hot spots were defined as areas in which Ki-67 staining was particularly higher relative to the adjacent areas. Usually, the invasive edge of the tumor was a hot spot. When a tumor had several hot spots, the “hottest” spot was selected as one of the three areas scored from the photographs (Fig 1). We aimed to count at least 500 cells in each case, as proposed by the International Ki67 Breast Cancer working group [8]. However, we were unable to count the required number in a case with low tumor cell density and small tumor size. The number of tumor cells counted in each photo ranged from 98 to 1453, and the total number counted on the three photos ranged from 375 to 2708. Fig 2 shows the distribution of Ki-67 LIs in the 493 cases.

Ki-67 was scored by two different methods: (1) the average method: manually counting the positive tumor cells in the three microphotos and calculating the average percentage of positive tumor cells; (2) the hot spot method: manually counting the positive tumor cells in one microphoto containing a hot-spot area. For tumors with visually homogenous Ki-67 staining, the highest Ki-67 LI of the three LIs was chosen. To express the heterogeneity of Ki-67 staining objectively, we also calculated the differences (Δ Ki-67) in Ki-67 LI between the average method and the hot spot method, and the ratio of the Ki-67 LI from the hot spot method to that from the average method (H/A ratio).

ΔKi−67=Ki−67 LI from hot spot method−Ki−67 LI from average method

$$\text{H}/\text{A}\;\text{ratio}=\frac{\text{Ki}-67\text{ LI from hot spot method}}{\text{Ki}-67\text{ LI from average method}}$$

### Clinicopathologic characteristics and survival data

Clinicopathologic characteristics and survival data were obtained by reviewing the electronic medical records, hematoxylin-eosin stained slides and immunohistochemically stained slides. Table 1 presents the clinicopathologic characteristics of the 493 analyzed breast cancers. All but two of the patients had received adjuvant hormonal therapy after surgery, and 65.7% had received adjuvant chemotherapy and 66.9% adjuvant radiation therapy. The median follow-up period was 5.8 years, ranging from 1.0 to 11.6 years, during which 23 patients had distant metastases and 5 had local recurrences as first events.

**Table 1:** Clinicopathologic characteristics of 493 primary luminal/HER-negative breast cancers.

Characteristic	Number of cases (%)
Age (yrs.)	
Median	50
Range	26–86
Sex	
Female	491 (99.6)
Male	2 (0.4)
Histologic subtype	
No special type	409 (83.0)
Lobular	32 (6.5)
Mucinous	26 (5.3)
Tubular	7 (1.4)
Cribriform	3 (0.6)
Others	16 (3.2)
Histologic grade	
I	171 (34.7)
II	217 (44.0)
III	105 (21.3)
pT stage	
pT1	315 (63.9)
pT2	168 (34.1)
pT3	7 (1.4)
pT4	3 (0.6)
pN stage	
pN0	297 (60.2)
pN1	146 (29.6)
pN2	30 (6.1)
pN3	16 (3.2)
Unknown	4 (0.8)
Tumor multiplicity	
Multiple	112 (22.7%)
Solitary	381 (77.3%)
Lymphovascular invasion	
Present	180 (36.5%)
Absent	313 (63.5%)
P53 expression	
Negative	444 (90.1%)
Positive	49 (9.9%)
Adjuvant hormonal therapy*	
Not done	2 (0.4%)
Done	485 (99.6%)
Adjuvant chemotherapy*	
Not done	167 (34.3%)
Done	320 (65.7%)
Adjuvant radiotherapy*	
Not done	161 (33.1%)
Done	326 (66.9%)

*Therapeutic information was not available for 6 patients due to loss of follow-up.

### Statistical analysis

Most statistical analyses were performed using SPSS version 19.0 (SPSS, Chicago, Illinois, USA). Receiver operating characteristic (ROC) curves analyses to determine optimal cutoff values of Ki-67 LIs, and ER/PR for predicting clinical outcomes, were processed using MedCalc version 16.1.2 (MedCalc Software bvba, Ostend, Belgium). The optimal cutoff values maximizing both sensitivity and specificity for predicting tumor recurrence were determined by reviewing the coordinates of ROC curves. Sensitivity, specificity, relative risk, 95% confidence intervals, and *p* values were calculated based on the cutoff values that had been chosen. Comparison ROC analysis proposed by DeLong et al. [18] was also performed using MedCalc. The significance of differences in Ki-67 LI obtained by the average method versus the hot spot method was calculated by paired t-tests. The correlation between Δ Ki-67 and Ki-67 LI determined by the two methods was analyzed by bivariate correlation analysis and presented as a Pearson correlation coefficient.

Kaplan-Meier survival curves for disease-free survival were plotted, and *p* values were calculated using log-rank tests. To identify independent prognostic factors, backward stepwise multivariate Cox regression analyses were carried out, employing covariates that were significantly associated with disease-free survival in univariate analysis. Hazard ratios (HR) and their 95% confidence intervals (CI) were calculated for each factor. *P* values were 2-tailed and *p* values < 0.05 were considered statistically significant.

## Results

### Comparison of Ki-67 LIs by the average method and the hot spot method

**Fig 3:**
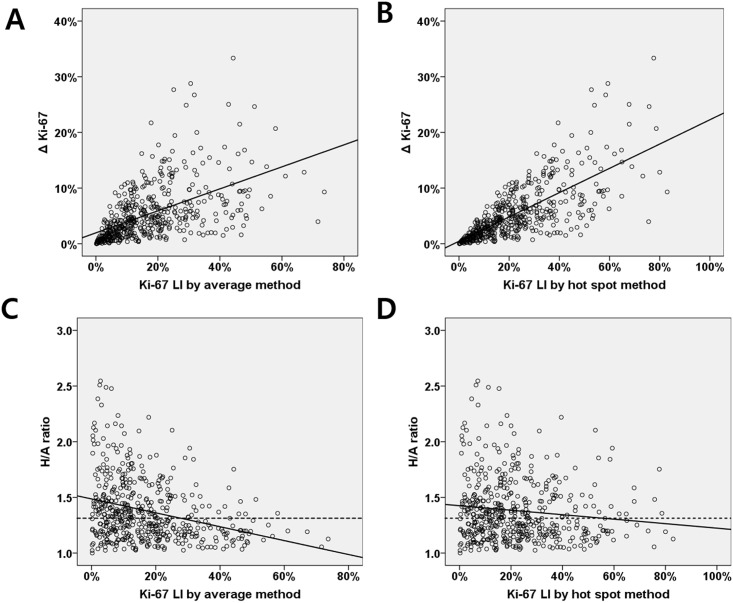
Correlation between Δ Ki-67, H/A ratio and Ki-67 labeling index measured by the two methods. (A, B) ΔKi-67 is moderately correlated with the Ki-67 labeling index (LI) based on the average method, and highly correlated with Ki-67 LI measured by the hot spot method (Pearson correlation coefficient, 0.535 and 0.731 respectively). (C, D) The H/A ratio is not correlated with Ki-67 LI measured by either method (Pearson correlation coefficients, -0.298 and -0.119, respectively).

We assessed Ki-67 LIs by the two methods. The median Ki-67 LI by the average method was 13.0% (IQR, 6.5%-23.6%), and that from the hot spot method was 18.5% (IQR, 9.0%-31.3%) (*p* <0.001; Fig 2). To estimate the intratumoral heterogeneity of Ki-67 expression, we calculated ΔKi-67 values and H/A ratios. The median ΔKi-67 values and H/A ratios were 3.9% (range, 0.01%-33.3%; IQR, 1.7%-7.1%) and 1.3 (range, 1.0–2.6; IQR, 1.2–1.5), respectively. When we evaluated the relationship between these heterogeneity indices and Ki-67 LIs (Fig 3), tumors with higher Ki-67 LIs by either method had significantly higher ΔKi-67 values (correlation coefficient, σ = 0.535, *p* <0.001, average method; σ = 0.731, *p* <0.001, hot spot method). However, H/A ratios were not correlated with the Ki-67 LIs obtained by either the hot spot method or the average method (correlation coefficients (σ): average method, -0.298 and hot spot method, -0.119)

### Prognostic impact of Ki-67 LIs: Average method vs. hot spot method

To compare the predictive performances of the Ki-67 LI obtained by the two methods for tumor recurrence, we carried out a ROC curve analysis (S1 Fig). The area under the curve (AUC) values of Ki-67 LI based on either method had moderate predictive power. The value from the hot spot method was slightly higher than from the average method, but the difference was not statistically significant (AUC: hot spot method, 0.711; average method, 0.700; *p* = 0.355). When the two methods were compared in early stage node-negative breast cancers, once again, there was no significant difference between them (AUC: hot spot method, 0.701; average method, 0.688; *p* = 0.706).

**Fig 4:**
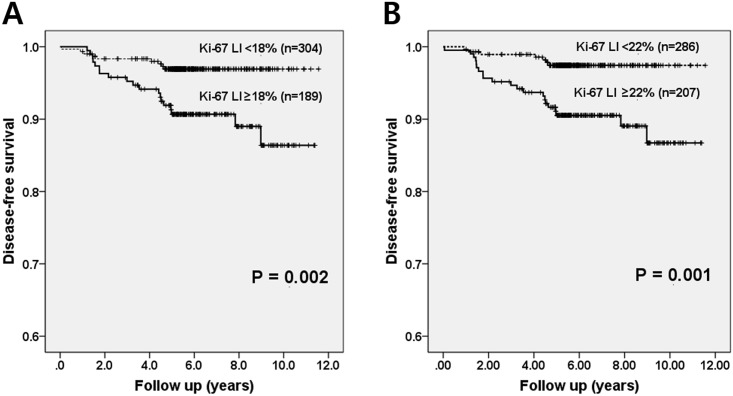
Kaplan-Meier survival curves of Ki-67 LI based on the average method and hot spot method. High Ki-67 labeling indices of tumors using a cutoff value of 18% in the average method (A) and 22% in the hot spot method (B) are associated with poor disease-free survival (*p* = 0.002, *p* = 0.001, respectively).

Based on the results of the ROC analysis, we were able to determine the optimal Ki-67 cutoff values for prognostication (Table 2). When tumors were assessed by the hot spot method, a cutoff of 22% was the most appropriate for predicting tumor recurrence, with sensitivity of 75.0% and specificity of 60.0%. Similarly, a Ki-67 cutoff of 18% was the most appropriate for the average method, with sensitivity of 67.9% and specificity of 63.4%. High expression of Ki-67 determined by these two cutoffs was associated with poor disease-free survival in Kaplan-Meier survival analysis (*p* = 0.002, average method; *p* = 0.001, hot spot method; Fig 4).

**Table 2:** Optimal Ki-67 cutoff values for predicting tumor recurrence.

Cutoff value*	Sensitivity	Specificity	Relative risk	95% CI	*P* value
Average method					
<17% vs ≥17%	67.86%	60.65%	1.7242	1.3047–2.2786	0.0001
<18% vs ≥18%	67.86%	63.44%	1.8561	1.4005–2.4599	< 0.0001
<19% vs ≥19%	60.71%	65.81%	1.7756	1.2848–2.4539	0.0005
Hot spot method					
<22% vs ≥22%	75.00%	60.00%	1.8750	1.4733–2.3862	< 0.0001
<23% vs ≥23%	64.29%	62.37%	1.7082	1.2656–2.3054	0.0005
<24% vs ≥24%	64.29%	64.52%	1.8117	1.3394–2.4506	0.0001

*Cutoff values were obtained based on ROC curve analyses.

Abbreviation: CI, confidence interval

### Variability of Ki-67 LIs measurements by the two methods

**Fig 5:**
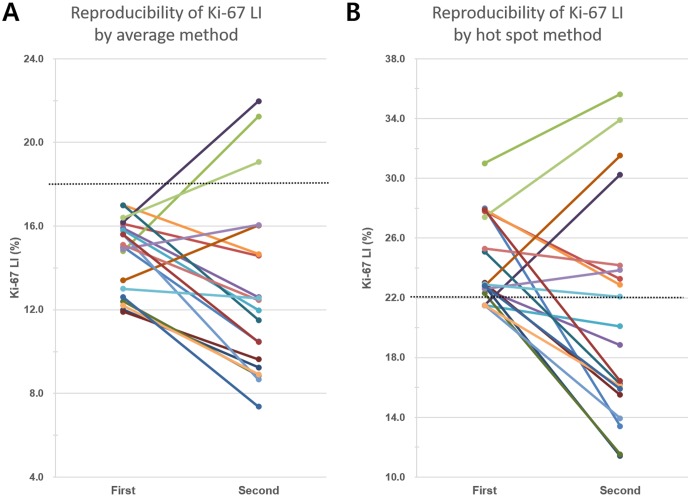
Variability of Ki-67 labeling index in repeated counts. (A) Twenty breast cancers that were initially classified into the low Ki-67 subgroup using the average method (cutoff value: 18%, dotted line) were re-counted by the same method. Only 3 cases were reclassified into the high Ki-67 subgroup in repeated count. (B) Of the 20 cases, 4 cases were initially classified into the low Ki-67 subgroup by the hot spot method (cutoff value: 22%, dot line). One of these was reclassified into the high Ki-67 subgroup, and 8 of the 16 cases originally classified into the high Ki-67 subgroup were reassigned to the low Ki-67 subgroup after a second count using the same method.

To estimate the reproducibility of the two methods, we re-selected three areas including one hot spot and then repeated Ki-67 LI measurement by both methods in 20 cases that had initial average Ki-67 LIs between 10% and 20% (S1 Table). All twenty cases had been initially classified into the low Ki-67 subgroup (Ki-67 LI <18%) by the average method (range of Ki-67 LI: 11.9%-17.0%). By the hot spot method, 16 of the 20 cases had been initially classified into the high Ki-67 subgroup (range of Ki-67 LI: 21.5%-31.0%). After re-counting, 3 cases were categorized into the high Ki-67 subgroup by the average method (Fig 5A). However, when using the hot spot method, 9 of the 20 cases were reclassified into different subgroups. One of the four cases that had been included in the low Ki-67 subgroup was reassigned to the high Ki-67 subgroup, and eight of the 16 cases that had been included in the high Ki-67 subgroup were reassigned to the low Ki-67 subgroup (Fig 5B).

### Prognostic value of ER and PR expression levels

We also analyzed the relationship between ER and PR expression levels and tumor recurrence. When 90% ER expression was used as the cutoff value, low ER expression was associated with tumor recurrence. Lower expression of PR was also associated with tumor recurrence using a cutoff value of 40% (S2 Fig). And low levels of ER and PR expression were associated with poor disease-free survival in Kaplan-Meier survival analysis (*p* = 0.036 and *p* = 0.006, respectively; S2 Fig).

### Multivariate analysis of disease-free survival

We performed a multivariate analysis of disease-free survival including pT stage, node metastasis, lymphovascular invasion, ER expression, PR expression and Ki-67 LI, which were significantly associated with disease-free survival in univariate analyses (Table 3). When we included Ki-67 LI obtained by the average method with a cutoff value of 18%, high T stage (HR, 3.397; 95% CI, 1.403–8.223; *p* = 0.007), nodal metastasis (HR, 4.024; 95% CI, 1.610–10.057; *p* = 0.003) and high Ki-67 LI (HR, 2431; 95% CI, 1.088–5.434; *p* = 0.030) were independent factors for poor clinical outcome. When we included Ki-67 LI from the hot spot method with a cutoff of 22%, high T stage (HR, 3.212; 95% CI, 1.319–7.823; *p* = 0.010), node metastasis (OR, 4.038; 95% CI, 1.614–10.100; *p* = 0.003) and high Ki-67 LI (OR, 3.058; 95% CI, 1.287–7.267; *p* = 0.011) were independent factors for poor clinical outcome (Table 4).

**Table 3:** Univariate analysis of factors associated with disease-free survival.

Variable		Hazard ratio	95% CI	P value
Age (years)	<50	1.000		
	≥50	0.987	0.471–2.071	0.973
Histologic grade	I & II	1.000		
	III	1.760	0.796–3.890	0.162
pT stage	pT1	1.000		
	pT2-4	5.651	2.401–13.301	<0.001
pN stage	pN0	1.000		
	pN1-3	5.718	2.317–14.111	<0.001
Tumor multiplicity	Single	1.000		
	Multiple	0.567	0.197–1.635	0.294
Lymphovascular invasion	Absent	1.000		
	Present	3.158	1.457–6.845	0.004
P53 overexpression	Absent	1.000		
	Present	1.122	0.339–3.716	0.851
Estrogen receptor	≥90%	1.000		
	<90%	2.185	1.036–4.611	0.040
Progesterone receptor	≥40%	1.000		
	<40%	2.756	1.303–5.828	0.008
Ki-67 index by average method	<18%	1.000		
	≥18%	3.301	1.491–7.309	0.003
Ki-67 index by hot spot method	<22%	1.000		
	≥22%	4.046	1.717–9.537	0.001
Chemotherapy	Not received	1.000		
	Received	1.466	0.622–3.456	0.382
Radiotherapy	Not received	1.000		
	Received	0.851	0.393–1.844	0.682

Abbreviation: CI, confidence interval

**Table 4:** Multivariate survival analysis using Cox proportional hazards regression models.

Model	Variable	Category	Hazard ratio	95% CI	*p* value
**1**	pT stage	T1 vs. T2-4	3.397	1.403–8.223	0.007
	pN stage	N0 vs. N1-3	4.024	1.610–10.057	0.003
	Lymphovascular invasion	Absent vs. present	1.097	0.462–2.608	0.834
	Estrogen receptor	≥90% vs. <90%	1.735	0.811–3.710	0.156
	Progesterone receptor	≥40% vs. <40%	1.900	0.886–4.075	0.099
	Ki-67 index by average method	<18% vs. ≥18%	2.431	1.088–5.434	0.030
**2**	T stage	T1 vs. T2-4	3.212	1.319–7.823	0.010
	N stage	N0 vs. N1-3	4.038	1.614–10.100	0.003
	Lymphovascular invasion	Absent vs. present	1.056	0.445–2.505	0.902
	Estrogen receptor	≥90% vs. <90%	1.634	0.764–3.494	0.205
	Progesterone receptor	≥40% vs. <40%	1.971	0.919–4.226	0.081
	Ki-67 index by hot spot method	<22% vs. ≥22%	3.058	1.287–7.267	0.011

P values were calculated from Cox proportional hazards regression models with backward stepwise selection.

Abbreviation: CI, confidence interval

## Discussion

Some breast cancers are heterogeneous in terms of Ki-67 expression with marked hot spots. This intratumoral heterogeneity is one of the obstacles that make the standardization of Ki-67 interpretation difficult. Up to now, how to count Ki-67 positive tumor cells in breast cancer with hot spots has been a contentious issue. In this study we compared the prognostic significance of Ki-67 LIs obtained by two methods the hot spot method and the average method, in hormone receptor-positive, HER2-negative breast cancers, to determine which of the methods had better sensitivity and specificity for predicting patients’ prognosis.

The choice of assessment method, the average method vs. hot spot method, did not generally affect the categorization of breast cancers into the high versus low Ki-67 subgroups. In the current study, only 26 (5.3%) of a total of 493 cases were classified into different subgroups (high vs. low Ki-67) when using the different counting methods. The Ki-67 LIs of these cases ranged from 12% to 18% in the average method, and 21% to 40% in the hot spot method (S2 Table). However, the ΔKi-67 (range, 2%-33%) and H/A ratio (range, 1.11–9.75) varied case by case. Therefore it seems that standardization of the counting method is most important for a minority of breast cancers whose Ki-67 LIs are between 10% and 20% in the average method, regardless of the extent of heterogeneity.

We calculated the cutoff value of Ki-67 LIs by ROC curve analysis. When the 18% and 22% cutoff values obtained by the average method and the hot spot method were applied, the sensitivity of the hot spot method was slightly higher than that of the average method (75.0% vs. 67.9%) while their specificities were similar (60.0% vs. 63.4%). In multivariate analysis, breast cancers with high Ki-67 LIs had poor disease-free survival, regardless of the assessment method. A few other studies have compared the average method with the hot spot method. Honma et al. reported that the Ki-67 index in the hottest spot was more useful than that determined by the average, since it independently predicted poor clinical outcomes [19]. However, they used empirical cutoff values such as 10% and 15% for the classification. Mu et al. found that the two counting methods were equally good predictors of tumor-free survival based on ROC analysis, though they recommended the hot spot method as it is more rapid [20]. However, they included hormone receptor (HR)-negative/HER2-positive and triple-negative breast cancers, and Ki-67 LI is not as effective in predicting prognosis for these subtypes as it is for luminal/HER2-negative breast cancers. In a case-control study, Arima et al. found that Ki-67 LIs assessed by hot spot counting with a 20% cutoff had the best predictive performance [21]. They compared the odds ratios from each method as a surrogate for prognostic power and showed that the hot spot method had a higher odds ratio and lower *p*-value, and therefore suggested that it was more reliable and effective than the average method.

In the current study, we analyzed ROC curves, just as Mu et al. [20] did, to compare the two methods more objectively. The AUC values of the two methods were similar as was their predictive performance. Which one should we then chose? Since consistency is one of the most important factors to be considered when standardizing a test, one has to choose the method that shows the better reproducibility. There have been some studies of the reproducibility of Ki-67 and the factors that influence it. Intratumoral heterogeneity, especially the existence of hot spots, has been viewed as one of the important factors that decrease the reproducibility of Ki-67 measurements. It was thought that counting only hot spot areas would reduce the discordance between observers [20, 21], because hot spots are literally areas that stand out clearly from their surroundings, and thus it would be more likely that pathologists would select similar areas. There have been only a few studies comparing the two methods with respect to this point, but most failed to yield results confirming this expectation.

A study by Shui et al. showed that the intraclass correlation coefficient (ICC) values of Ki-67 obtained by the hot spot method were similar to those obtained by the average method (ICC of hot spot method, 0.894; ICC of average method, 0.904) [11]. In another study Varga et al. demonstrated that the average method had better intra- and inter-observer reliabilities than counting hot spots [10], and we obtained a similar result in a previous study: when we compared the inter-observer variability of the Ki-67 indices obtained by the hot spot and average methods in cases with hot spots, we found no difference between the ICC values obtained (0.737 vs. 0.736) [22]. Recently, Leung and his colleagues reported the results of an international multicenter study of Ki-67 reproducibility [23]. They counted Ki-67 positive tumor cells in three different ways: un-weighted global, weighted global and ‘hot spots only’. They found that only the un-weighted global method (ICC, 0.87) met the specified criteria for reproducible scoring.

Although there had been some differences in the sample platform, counting methods as well as criteria for scoring, the above observations do suggest in common that counting hot spots is not a robust or reliable option for Ki-67 counting. Currently, there exists no solid evidence that the hot spot method is superior to the average method in terms of reproducibility. In this study, we re-scored Ki-67 LIs in 20 breast cancers that had Ki-67 LIs around the cutoff value, and we found that the reproducibility of the hot spot method was much poorer than that of the average method. This result could be attributed to the following factors. First, defining hot spots in whole sections may be quite subjective: there is no numerical value that defines a hot spot; we simply assume them to be areas with a higher density of Ki-67-positive cells than the surrounding areas. Therefore, there exists intra-observer and inter-observer variability in defining hot spots. A minor difference in the selection of a hot spot can make a significant difference in Ki-67 score [23]. In our study, there was a difference of >10% between the first and second counts in 4 of the 20 cases (S1 Table). Second, there may be multiple hot spots with different Ki-67 LIs in a single tumor section.

Thus, we suggest using the average method rather than the hot spot method; for the time being, counting multiple random selected areas including hot spot appears to be a more robust and reliable approach though it shows a slightly lower hazard ratio for predicting tumor recurrence than the hot spot method. Further studies are warranted to establish an appropriate counting method for Ki-67 that is more predictive and more reproducible.

We also evaluated the prognostic value of hormone receptor expression levels. Low ER (<90%) and PR (<40%) expression levels were associated with poor disease-free survival in our patients. Many previous studies have shown that PR expression is a good prognostic biomarker in luminal breast cancers [24–26] and it is also a marker distinguishing luminal A from luminal B subtypes [27, 28]. The 2013 St. Gallen Consensus also proposed low or negative PR expression as a surrogate for defining luminal B cancers [5], based on the work of Prat et al. [27]. The authors from the latter study used an empirical cutoff of >20% of PR-positive tumor cells and demonstrated significant survival differences within IHC-based luminal A tumors [27]. Like Ki-67, PR expression is a continuous parameter and can show intratumoral heterogeneity. Therefore, defining a cutoff value for low PR expression is difficult, as it is for Ki-67. To be able to use PR expression as a prognostic marker in daily practice, more studies aimed at defining the appropriate cutoff value appear essential.

In conclusion, we have shown that both the hot spot method and average method have good predictive ability for tumor recurrence in luminal/HER2-negaitve breast cancers, but the average method is more reproducible. We suggest using the average method at this point in time.

## Supporting information

S1 Fig Comparison of the predictive powers of Ki-67 labeling index based on the average method and the hot spot method using comparison ROC analysis. The values of the areas under the curve (AUC) obtained by the two methods were similar (average method, 0.700; hot spot method, 0.711; *p* = 0.355).
(TIF)

S2 Fig ROC curves for assessing the cutoff values of percent ER and PR positivity, and Kaplan-Meier survival curves based on them. The areas under curve for ER (A) and PR (B) were 0.612 and 0.602, respectively using a 90% cutoff value for ER and 40% for PR. Lower expression of ER (C, <90%) and PR (D, <40%) was correlated with shorter disease-free survival time (*p* = 0.036 and *p* = 0.006, respectively).
(TIF)

S1 Table Variability of Ki-67 labeling indices in a repeated count. (DOCX)

S2 Table Twenty-six cases which were classified to different groups using a different counting method. (DOCX)
