# Supplementary figures and images for: A comparison of Ki-67 counting methods in luminal Breast Cancer: The Average Method vs. the Hot Spot Method
Source: PLoS One. 2017 Feb 10;12(2):e0172031. doi: 10.1371/journal.pone.0172031 (PMC5302792; doi:10.1371/journal.pone.0172031)

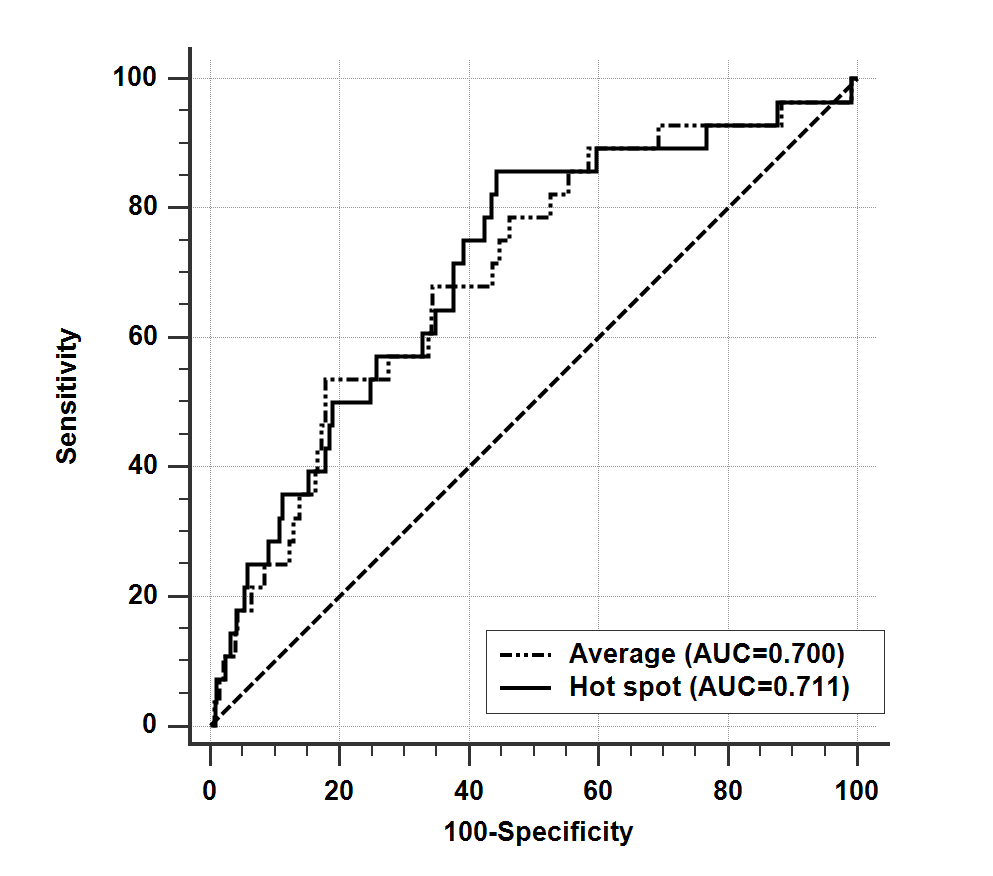

Supplement: S1 Fig — The values of the areas under the curve (AUC) obtained by the two methods were similar (average method, 0.700; hot spot method, 0.711; p = 0.355). (TIF) [file pone.0172031.s001.tif]

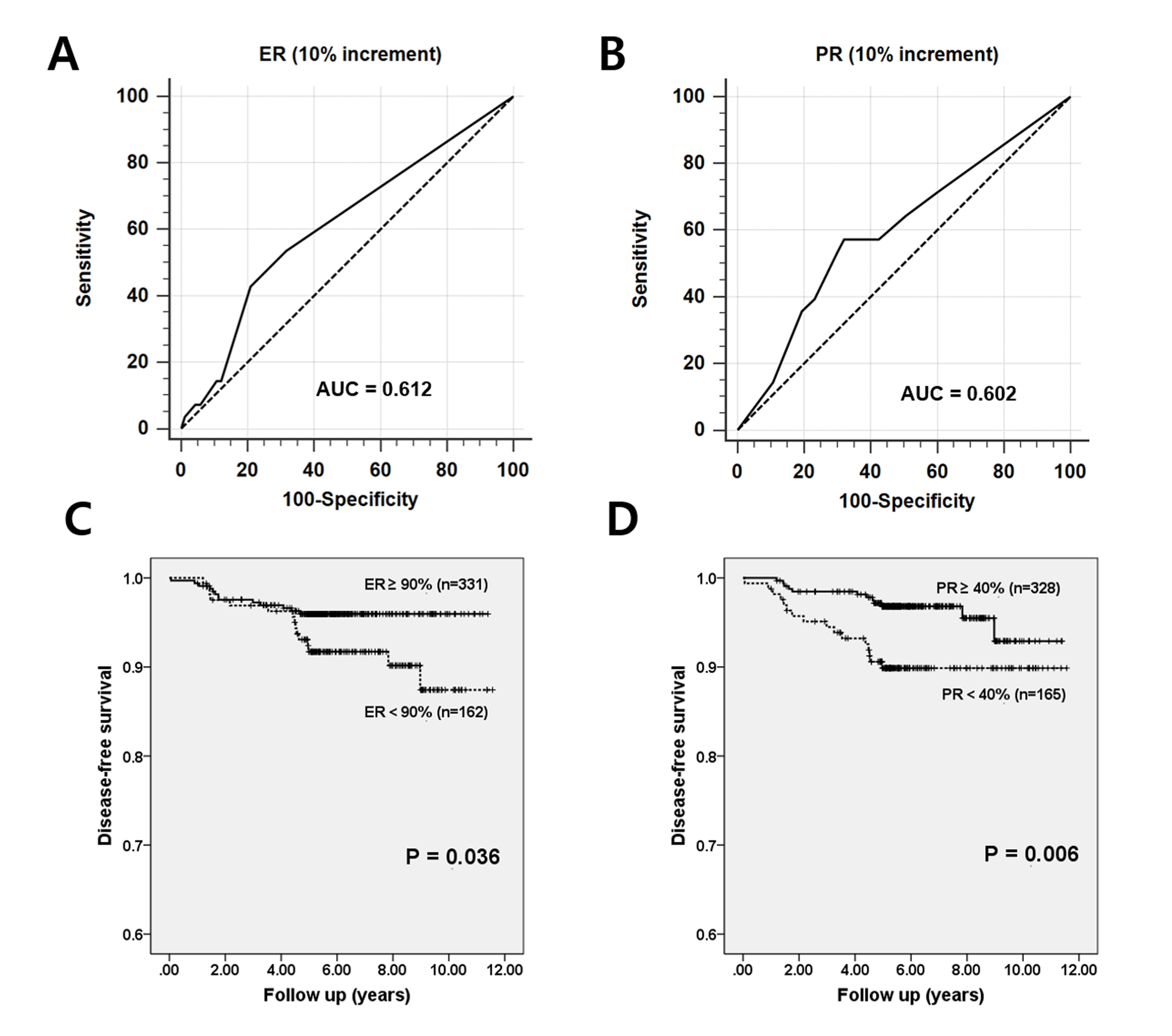

Supplement: S2 Fig — The areas under curve for ER (A) and PR (B) were 0.612 and 0.602, respectively using a 90% cutoff value for ER and 40% for PR. Lower expression of ER (C, <90%) and PR (D, <40%) was correlated with shorter disease-free survival time (p = 0.036 and p = 0.006, respectively). (TIF) [file pone.0172031.s002.tif]
