# Supplementary material for: A comparison of Ki-67 counting methods in luminal Breast Cancer: The Average Method vs. the Hot Spot Method
Source: PLoS One. 2017 Feb 10;12(2):e0172031. doi: 10.1371/journal.pone.0172031 (PMC5302792; doi:10.1371/journal.pone.0172031)
Supplement: S1 Table — (DOCX) [file pone.0172031.s003.docx]

**S1 Table. Variability of Ki-67 labeling indices in a repeated count**

| Case No. | Ki-67 LI by average method | | | Ki-67 LI by hot spot method | | |
| --- | --- | --- | --- | --- | --- | --- |
|  | First counting (%) | Second counting (%) | Concordance of grouping between two counts* | First counting (%) | Second counting (%) | Concordance of grouping between two counts** |
| 10 | 15.0 | 10.5 | Concordant | 28.0 | 13.4 | Discordant |
| 51 | 16.1 | 14.6 | Concordant | 27.8 | 23.3 | Concordant |
| 56 | 14.8 | 21.2 | Discordant | 31.0 | 35.6 | Concordant |
| 80 | 15.9 | 12.6 | Concordant | 22.6 | 18.8 | Discordant |
| 106 | 15.8 | 12.0 | Concordant | 21.5 | 20.1 | Concordant |
| 119 | 17.0 | 14.7 | Concordant | 27.9 | 22.9 | Concordant |
| 121 | 12.0 | 9.2 | Concordant | 22.8 | 11.4 | Discordant |
| 163 | 11.9 | 9.6 | Concordant | 23.0 | 15.5 | Discordant |
| 178 | 12.4 | 8.8 | Concordant | 22.3 | 11.5 | Discordant |
| 204 | 16.2 | 22.0 | Discordant | 21.5 | 30.3 | Discordant |
| 205 | 17.0 | 11.5 | Concordant | 25.1 | 16.2 | Discordant |
| 220 | 13.4 | 16.0 | Concordant | 22.8 | 31.5 | Concordant |
| 335 | 15.6 | 8.7 | Concordant | 21.5 | 13.9 | Concordant |
| 378 | 15.1 | 12.5 | Concordant | 25.3 | 24.2 | Concordant |
| 387 | 16.4 | 19.1 | Discordant | 27.4 | 33.9 | Concordant |
| 406 | 14.9 | 16.1 | Concordant | 22.6 | 23.9 | Concordant |
| 415 | 13.0 | 12.6 | Concordant | 22.9 | 22.1 | Concordant |
| 447 | 12.2 | 8.9 | Concordant | 21.5 | 16.1 | Concordant |
| 466 | 12.6 | 7.4 | Concordant | 22.8 | 15.9 | Discordant |
| 485 | 15.6 | 10.5 | Concordant | 27.9 | 16.4 | Discordant |

*based on the cutoff value of 18%; **based on the cutoff value of 22%

Abbreviation: LI, labeling index
