# Supplementary material for: A comparison of Ki-67 counting methods in luminal Breast Cancer: The Average Method vs. the Hot Spot Method
Source: PLoS One. 2017 Feb 10;12(2):e0172031. doi: 10.1371/journal.pone.0172031 (PMC5302792; doi:10.1371/journal.pone.0172031)
Supplement: S2 Table — (DOCX) [file pone.0172031.s004.docx]

**S2 Table. Twenty-six cases which were classified to different groups using a different counting method**

| Case No. | Average method | | Hot spot method | |
| --- | --- | --- | --- | --- |
|  | Ki-67 LI (%) | Group* | Ki-67 LI (%) | Group** |
| 7 | 15.6 | Low | 26.0 | High |
| 10 | 15.0 | Low | 28.0 | High |
| 51 | 16.1 | Low | 27.8 | High |
| 56 | 14.8 | Low | 31.0 | High |
| 60 | 17.9 | Low | 28.8 | High |
| 80 | 15.9 | Low | 22.6 | High |
| 119 | 17.0 | Low | 27.9 | High |
| 121 | 12.0 | Low | 22.8 | High |
| 124 | 17.2 | Low | 25.5 | High |
| 163 | 11.9 | Low | 23.0 | High |
| 178 | 12.4 | Low | 22.3 | High |
| 180 | 20.7 | High | 21.7 | Low |
| 199 | 17.9 | Low | 22.6 | High |
| 205 | 17.0 | Low | 25.1 | High |
| 241 | 20.6 | High | 21.9 | Low |
| 300 | 18.3 | High | 21.0 | Low |
| 378 | 15.1 | Low | 25.3 | High |
| 387 | 16.4 | Low | 27.4 | High |
| 406 | 14.9 | Low | 22.6 | High |
| 415 | 13.0 | Low | 22.9 | High |
| 421 | 19.7 | Low | 20.9 | High |
| 464 | 17.8 | Low | 39.5 | High |
| 465 | 17.8 | Low | 24.4 | High |
| 466 | 12.6 | Low | 22.8 | High |
| 485 | 15.6 | Low | 27.9 | High |
| 488 | 17.6 | Low | 25.6 | High |

*based on the cutoff value of 18%;** based on the cutoff value of 22%

Abbreviation: LI, labeling index
